# Guillain-Barre syndrome and pulmonary embolism in an adult female with COVID-19 infection in Ghana: A case report

**DOI:** 10.1097/MD.0000000000033754

**Published:** 2023-05-17

**Authors:** Eugene Tetteh-Wayoe, Fiifi Duodu, Prince Kwabla Pekyi-Boateng, Nana Boakye Agyeman Badu, Albert Akpalu, Patrick Adjei

**Affiliations:** a Department of Medicine and Therapeutics, Korle-Bu Teaching Hospital, Accra, Ghana; b Department of Medicine and Therapeutics, University of Ghana Medical School, Accra, Ghana.

**Keywords:** COVID-19, Guillain-Barre syndrome, neuropathy, paralysis, Sub-Saharan Africa

## Abstract

The coronavirus disease 2019 (COVID-19) pandemic began at the end of 2019 in Wuhan, the capital of Hubei Province, China. This novel coronavirus is classified as severe acute respiratory syndrome coronavirus 2 (SARS-CoV-2). Neurological manifestations are commonly associated with moderate to severe COVID-19 infection. Guillain-Barré syndrome (GBS) is a rare immune-mediated postinfectious neuropathy but there has been an increase in the number of cases of GBS associated with COVID-19, supporting the present body of global evidence of the notable association between the 2 conditions. We present the first proven case of GBS and pulmonary embolism associated with COVID-19 infection in Ghana, West Africa.

**Case Presentation:**

A 60-year-old apparently healthy female presented in August 2020 to the COVID-19 treatment center of the Korle-Bu Teaching Hospital in Accra, Ghana from a referral facility following a week’s history of low-grade fever, chills, rhinorrhoea, and generalized flaccid limb weakness. A positive SARS-CoV-2 test result was recorded 3 days after the onset of symptoms and the patient had no known chronic medical condition. Following cerebrospinal fluid analysis, neurophysiological studies and a chest computed tomography pulmonary angiogram, Guillain-Barre syndrome and pulmonary embolism were confirmed. The patient was however managed supportively and then discharged after 12 days on admission, as he made mild improvement in muscular power and function.

**Conclusion:**

This case report adds to the body of evidence of the association between GBS and SARS-CoV-2 infection, particularly from West Africa. It further highlights the need to anticipate potential neurological complications of SARS-CoV-2, particularly GBS even in mild respiratory symptoms for prompt diagnosis and initiation of appropriate therapy to improve outcomes and avert long-term deficits.

## 1. Introduction

Guillain-Barre syndrome (GBS) represents the most common cause of acute flaccid paralysis.^[1]^ The classic manifestation is an immune-mediated acute onset demyelinating polyradiculoneuropathy (acute inflammatory demyelinating polyneuropathy) typically presenting with ascending weakness, loss of deep tendon reflexes, and sensory deficits. Well-known causative pathogens include bacteria (e.g., *Campylobacter jejuni, Mycoplasma pneumoniae*). Viruses such as cytomegalovirus, Epstein–Barr virus, influenza virus, hepatitis E virus, and recently Zika virus have been also reported.^[1,2]^ Following the first reported case of coronavirus disease 2019 (COVID-19)-associated GBS in Wuhan, China,^[3]^ there has been extensive case reportage^[1]^of this new entity further supporting the evidence of the association. This case adds to the reportage as the first proven case of COVID-19-related GBS with pulmonary embolism in Ghana since the confirmation of the first 2 cases in March 2020. The paper will discuss the major clinical and laboratory findings and elucidate possible underlying pathophysiology for the case features therein.

## 2. Case presentation

A retired, 60-year-old female who was apparently healthy, presented in August 2020 to the COVID-19 treatment center of the Korle-Bu Teaching Hospital in Accra from a referral facility. She had a week’s history of low-grade fever, chills, rhinorrhoea, and generalized limb weakness. The patient had tested positive for COVID-19 infection 3 days following the onset of symptoms. There was no history of cough, pleuritic chest pain, exertional dyspnea or dyspnea at rest, recent calf pain/swelling, prolonged bedrest, long-distance travel and cancer diagnosis in the last 6 months. The generalized weakness was of sudden-onset and progressive, initially involving the lower limbs and extending to the upper limbs. There was no associated bowel or bladder incontinence and no sensory deficit. Her past medical history was negative for hypertension, diabetes or asthma.

The patient on presentation was not acutely ill-looking but febrile: 38.2 C, not clinically pale, anicteric, acyanosed, and well-hydrated. There was no palpable lymphadenopathy. A neurological exam revealed a conscious and alert woman with no cranial nerve deficits. She had hypotonia, areflexia and zero power in all limbs (Medical Research Council, MRC - 0/5). The plantar response was absent bilaterally. No sensory level was elicited. The pulse rate was 96 beats per minute with a regular rhythm, normal precordial findings and the blood pressure reading was 164/94 mm Hg. The respiratory rate was 20 cycles per minute with oxygen saturation of 98% on room air and normal chest findings. The abdominal exam was essentially normal whiles fasting blood glucose reading was 9.1 mmol/L.

Lumbar puncture for cerebrospinal fluid (CSF) analysis, neurophysiological studies, chest computed tomography pulmonary angiogram confirmed Guillain-Barre syndrome and pulmonary embolism (Tables 1–3).

**Table 1:** 

Cerebrospinal fluid biochemistry		Normal range
Appearance	Clear and colourless	
Glucose	7.5 mmol/L	2.5–4. O mmol/L
Albumin	105 mg/dL	10–30 mg/dL
Globulin	25.7 mg/dL	3.7- 5.7mg/dL
CSF IgG index	0.67	0.3–0.7
CSF IgG/albumin ratio	0.24	0.05–0.27

Evidence of cerebrospinal fluid albumin cytologic dissociation with elevated globulin.

CSF = cerebrospinal fluid.

**Table 2:** 

Cerebrospinal fluid bacteriology			Normal range
Macroscopy	Appearance	Clear and colorless	
Microscopy	WBC Count	5*10 ^6/L	0-5*10 ^6/L
	RBC Count		
	Gram stain	No organism seen	
CULTURE		No bacterial growth	

RBC = red blood cell, WBC = white blood cell.

**Table 3:** Neurophysiologic study findings.

Table 3A Motor nerve conduction:					
Nerve	Position	Latency [ms]	Amplitude [mV]	Distance [mm]	CV [m/s]
Median- Abductor pollicis brevis- right	Wrist	Absent	Absent	Absent	Absent
	Elbow	Absent	Absent	Absent	Absent
Ulnar- adductor digiti minimi- Left	Wrist	Absent	Absent	Absent	Absent
	B. Elbow	Absent	Absent	Absent	Absent
Tibial- adductor hallucis (knee)-right	Ankle	Absent	Absent	Absent	Absent
	Knee	Absent	Absent	Absent	Absent
Table 3B Sensory nerve conduction:					
Nerve	Position	Latency [ms]	Amplitude [mV]	Distance [mm]	CV [m/s]
Median- digit II-right	Wrist	5.6	3.0	140	25
Ulnar- digit V- left	Wrist	Absent	Absent	Absent	Absent
Table 3C F–wave:					
Nerve	M-Latency [ms]	M-amplitude [mV]	Fmin [ms]	F-M [ms]	F/ M
Median- adductor digiti minimi- right	Absent	Absent	Absent	Absent	Absent
Ulnar- adductor digiti minimi- left	Absent	Absent	Absent	Absent	Absent
Tibial- adductor hallucis (knee)- right	Absent	Absent	Absent	Absent	Absent
Table 3D Electromyography:					
Muscle	Notes				
Abductor digiti minimi- right	Abnormal—positive sharp waves and fibrillation potentials, motor unit action potential morphology and recruitment not assessed				
Abductor pollicis brevis- right	Abnormal—positive sharp waves and fibrillation potentials, motor unit action potential morphology and recruitment not assessed				

### 2.1. Exam findings and diagnostic impression

The nerve conduction study shows electrodiagnostic evidence suggestive of absent motor responses in all the motor nerves evaluated in the upper and lower limbs. The median sensory nerve shows prolonged onset latency, reduced (Sensory nerve action potential) SNAP amplitude and slowed conduction velocity on the Right. All F-Wave latencies were absent. There was evidence of muscle electrical instability in the muscles evaluated in the upper limbs.

## 3. Chest computed tomogram And pulmonary angiogram

Both lung fields are well aerated and showed normal computed tomogram features. No masses or pleural effusion seen bilaterally. Bilateral segmental pulmonary emboli with small wedge-shaped right pulmonary opacity.

## 4. Head computed tomogram scan

Linear hypodensity in the anterior limb of the left internal capsule likely a small chronic infarct.

## 5. Clinical progress and outcome

Patient’s condition over the period of admission remained relatively stable. She however had labile blood pressure readings ranging between 164/94 mm Hg and 233/123 mm Hg but no respiratory distress or swallowing difficulty and as such intensive care was not initiated. Her quadriplegia improved slightly about 6 days into admission with power in the upper limbs increasing to 2 (MRC 2/5) with regular intensive physiotherapy sessions. Her blood pressures were managed with oral amlodipine 10 mg od, lisinopril 10 mg od and methyldopa 250 mg bid. The newly diagnosed diabetes was managed with oral metformin 1 g bid and gliclazide 30 mg od with resultant controlled blood glucose profile over the period of admission. She was also put on oral Rivaroxaban (Xarelto) 15 mg bd for the pulmonary embolism. Patient’s repeat nasopharyngeal swab for SARS-CoV 2 PCR test 10 days post admission recorded negative and as such discharged to continue physiotherapy and rehabilitation on out-patient basis. Upper and lower limb muscle power was MRC 2/5 upon discharge.

**Figure 1.**
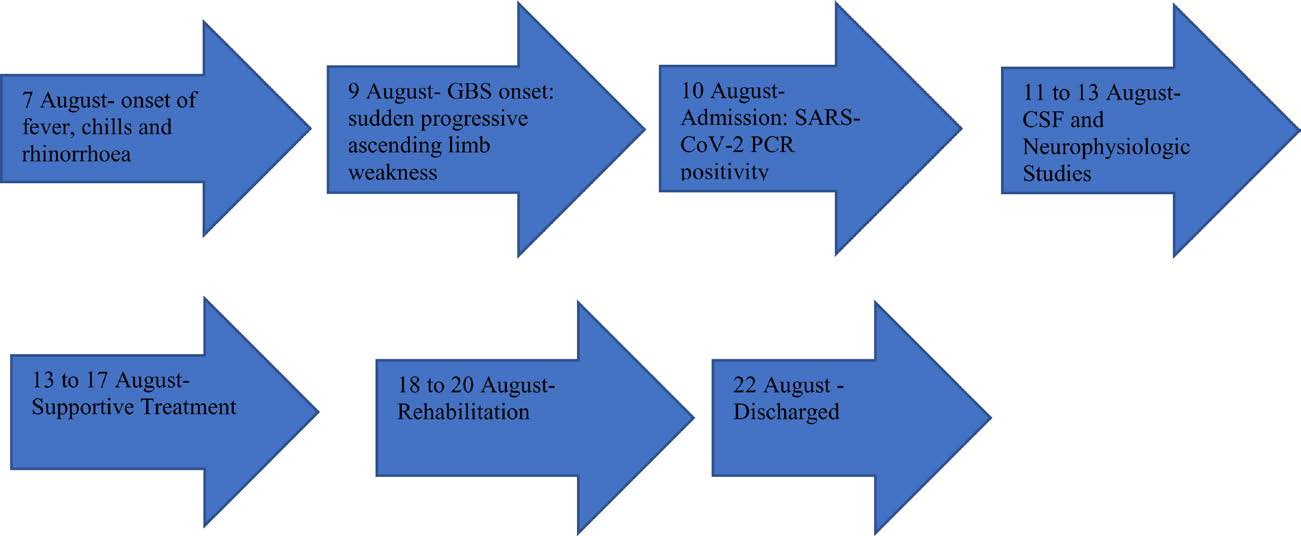
Timeline of clinical events, diagnosis, and outcomes.

Patient was pleased with the prompt manner of diagnostic investigations and efficient nature of treatment offered to ensure the best possible outcome. She remained elated for the degree of recovery achieved with her overall management. The time of specific clinical events, diagnosis and outcomes are available at Figure 1.

### 5.1. Diagnoses

Guillain-Barre Syndrome; Acute motor sensory axonal variant

Pulmonary Embolism.

### 5.1. Differential diagnosis

Parainfectious myositis.

The clinical assessment saw significant improvement in motor and sensory function after day 6 on admission. Patient-assessed outcomes saw gradual improvement after day 6 on admission until she was discharged. No follow-up diagnostic tests were conducted. No major intervention was performed as such, no tolerability had to be assessed. No adverse and unanticipated events were also recorded.

## 6. Discussion

The first reported case of COVID-19-associated GBS was reported from China as a suspected parainfectious disease, as the patient developed COVID-19 symptoms 7 days after the onset of GBS symptoms.^[3]^

The notable epidemic of severe acute respiratory syndrome that arose from SARS-COV in Asia in February 2003^[4]^ was mainly characterized by myalgia, fever, and other systemic symptoms from which patients usually recovered after a few days.^[5]^ Neurological manifestations were observed in 30% of the patients during the outbreak.^[6]^

SARS-COV 2 related GBS spectrum disorder has seen increasing reportage globally since the onset of the pandemic and several case reports have already been published thus far,^[1]^ but none from Sub-Saharan region. GBS is an acute onset demyelinating polyneuropathy with ascending paralysis. The major subtypes of GBS include acute inflammatory demyelinating polyneuropathy, acute motor axonal neuropathy, acute motor sensory axonal neuropathy, and Miller Fisher syndrome. Diagnosis of GBS relies on the results of clinical, electrophysiological, and CSF examinations (classically albuminocytologic dissociation).^[1,2,7]^

The mechanism of the GBS occurrence is based on molecular mimicry and anti-ganglioside antibodies during or following a susceptible infection in genetically predisposed patients.^[8]^ These antibodies portray the strongest association with certain forms of GBS.^[9,10]^ A likely mechanism is an autoimmune reaction in which the antibodies on the pathogen, which are similar to the protein structures of the peripheral nerve components, cause damage to the nervous system.^[11]^ This similarity has been termed “molecular mimicry” which is defined as the theoretical likelihood that sequence similarities between foreign and self-peptides are enough to lead to the cross-activation of autoreactive B cell or T cell by pathogen-derived peptides.^[8]^

Our case is the first patient in Ghana during this COVID-19 pandemic with GBS in SARS-CoV2 infection also likely as a parainfectious process and presented with mild respiratory symptoms and ascending paralysis. This patient tested negative for nasopharyngeal swab reverse transcriptase polymerase chain reaction (RT-PCR) SARS-CoV2 14 days after the first positive test. Neurological presentations of COVID-19 are often associated with severe acute respiratory distress syndrome but our patient presented with a neurological complication following mild COVID-19 symptoms. The timeline of GBS development is usually 1 to 2 weeks after an underlying infection and most cases of COVID-19 related GBS have been reported between 1- and 4-weeks post infection.^[1]^ The onset of ascending weakness with mild respiratory symptoms and the confirmation of RT-PCR SARS - CoV-2 3 days thereafter in our patient is quite atypical. This however shows similarity with 2 reported cases of GBS diagnosed with or before the confirmation of SARS-CoV 2 by RT-PCR.^[3,12]^

Of diagnostic significance, the CSF examination showed normal cell counts, increased albumin and elevated CSF/serum albumin ratio. These results give an albumin cytologic dissociation profile, the characteristic finding in GBS as shown in Table 1. This important CSF exam result is consistent with most reported cases of COVID-19 related GBS and particularly, in a systematic review of 52 case series involving 73 patients with GBS, CSF analysis detected albumin cytological dissociation (cell count < 5/µL with elevated CSF proteins) in 71.2% of the cases (42 out of 59 cases with full CSF results) with a median CSF protein of 100.0 mg/dL (min: 49, max: 317 mg/dL).^[1]^ Cerebrospinal fluid RT-PCR for severe acute respiratory syndrome coronavirus (SARS-CoV-2) RNA was however not performed in our patient and as such difficult to rule out a possible direct central nervous viral invasion or involvement. The other supportive laboratory results are shown in (Table 4) and notably highlighted as deranged are the white cell count erythrocyte sedimentation rate, D-Dimers and hemoglobin A1C.

**Table 4:** Results of laboratory tests and investigations conducted.

Full blood count	Liver function test	Renal function tests	Lipid profile	Glycosylated haemoglobin
Haemoglobin- 12 g/dL	Total Bilirubin- 3 umol/L	Sodium- 141 mmol/L	Total cholesterol- 5.42 mmol/L	DCCT- 7.9% IFCC- 62.8 mmol/mol
Platelet count-358*10^9/L	Direct Bilirubin- 1.6 umol/L	Potassium- 4.9mmol/L Chloride- 101 mmol/L	Triglyceride- 1.96 mmol/L	
White cell Count-13.93*10 ^9/L Neutrophil # -10.10*10 ^9/L	AST- 35 U/L ALT- 54 U/L ALP- 121U/L GGT- 67 U/L	Urea- 5.5 mmol/L Creatinine- 79 umol/L	HDL- 1.2 mmol/L LDL- 3.32 mmol/L	
Erythrocyte sedimentation rate- 65 mm fall/hour (4–7)	Total Protein- 66 g/L Albumin- 49 g/L		D-dimers- 0.72ug/L (< 0.5)	

Neurophysiological studies were successfully performed in this index case and the findings were consistent with acute motor sensory axonal variant GBS (Table 3). Regarding the Brighton criteria for diagnostic certainty, rating can be placed at level 1 (consistent clinical features, typical CSF results and nerve conduction studies).^[13,14]^ In addition, the finding of absent motor responses in this case may be suggestive of presumed distal demyelination with unexcitable nerves. This type of pathology has been reported in GBS with anti GM1 antibodies^[15]^ which ideally should have been tested for in this index case but regrettably not done due to time and resource constraints. This test would have further highlighted the systemic inflammatory nature of SARS-CoV-2 infection with prognostic implications to the development of chronic inflammatory demyelinating polyneuropathy.^[16]^

An interesting and important finding in our patient was the presence of pulmonary emboli with relatively normal lung parenchyma as indicated above. The absence of any history of pulmonary emboli or predisposition to venous thromboembolism makes this pathological finding a part of the COVID-19 sequelae. Thromboembolism due to vasculopathy and coagulopathy induced by SARS-CoV2 infection has been well described and reported.^[17]^

It is also worth highlighting that, dysautonomia, a well-recognized feature of GBS, characterized our patient’s clinical course and was evidenced mainly by new onset elevated labile blood pressure readings for which intensive antihypertensive regimen had to be employed to attain control. New onset moderate hyperglycemia was also observed, which responded well to oral antidiabetic agents as stated above.

## 7. Conclusion

This case report adds to the body of evidence of the association between GBS and SARS-CoV-2 infection particularly from West Africa. It also supports the increasing global reportage of COVID-19 associated neuropathies and related sequelae. It is imperative to anticipate potential neurological complications of SARS-CoV-2 particularly GBS even in mild respiratory symptoms for prompt diagnosis and initiation of appropriate therapy to improve outcomes and avert long-term deficits.

## Author contributions

**Conceptualization:** Eugene Tetteh-Wayoe, Fiifi Duodu, Nana Boakye Agyeman Badu, Patrick Adjei.

**Data curation:** Eugene Tetteh-Wayoe, Fiifi Duodu, Albert Akpalu.

**Formal analysis:** Eugene Tetteh-Wayoe, Fiifi Duodu, Patrick Adjei.

**Investigation:** Eugene Tetteh-Wayoe, Nana Boakye Agyeman Badu, Albert Akpalu, Patrick Adjei.

**Methodology:** Fiifi Duodu, Prince Kwabla Pekyi-Boateng.

**Resources:** Eugene Tetteh-Wayoe.

**Software:** Albert Akpalu.

**Supervision:** Nana Boakye Agyeman Badu.

**Validation:** Fiifi Duodu, Albert Akpalu, Patrick Adjei.

**Writing – original draft:** Eugene Tetteh-Wayoe.

**Writing – review & editing:** Eugene Tetteh-Wayoe, Fiifi Duodu, Prince Kwabla Pekyi-Boateng, Nana Boakye Agyeman Badu, Albert Akpalu, Patrick Adjei.

## References

[1] Leonhard SE Mandarakas MR Gondim FAA. Diagnosis and management of Guillain–Barré syndrome in ten steps. Nat Rev Neurol. 2019;15:671–83. 31541214 10.1038/s41582-019-0250-9 PMC6821638

[2] Willison HJ Jacobs BC van Doorn PA. Guillain-Barré syndrome. The Lancet. 2016;388:717–27. 10.1016/S0140-6736(16)00339-1 26948435

[3] Zhao H Shen D Zhou H. Guillain-Barré syndrome associated with SARS-COV-2 infection: causality or coincidence?. Lancet Neurol. 2020;19:383–4. 32246917 10.1016/S1474-4422(20)30109-5 PMC7176927

[4] Rainer TH Lee N Ip M. Features discriminating SARS from other severe viral respiratory tract infections. Eur J Clin Microbiol Infect Diseases. 2007;26:121–9. 17219094 10.1007/s10096-006-0246-4 PMC7088160

[5] Leung TW Wong KS Hui AC. Myopathic changes associated with severe acute respiratory syndrome: a postmortem case series, archives of neurology. U.S. National Library of Medicine. no date. Available at: https://pubmed.ncbi.nlm.nih.gov/16009768/. [access date March 15, 2023]. 10.1001/archneur.62.7.1113 16009768

[6] Lee N Hui D Wu A. A major outbreak of severe acute respiratory syndrome in Hong Kong. N Engl J Med. 2003;348:1986–94. 12682352 10.1056/NEJMoa030685

[7] Kieseier BC Mathey EK Sommer C. Immune-mediated neuropathies. Nat Rev Dis Primers. 2018;4. 10.1038/s41572-018-0027-2 30310069

[8] Wim Ang C Jacobs BC Laman JD. The guillain–barré syndrome: a true case of molecular mimicry. Trends Immunol. 2004;25:61–6. 15102364 10.1016/j.it.2003.12.004

[9] Ariga T Miyatake T Yu RK. Recent studies on the roles of antiglycosphingolipids in the pathogenesis of neurological disorders. J Neurosci Res. 2001;65:363–70. 11536318 10.1002/jnr.1162

[10] Rivera-Correa J de Siqueira IC Mota S. Anti-ganglioside antibodies in patients with Zika virus infection-associated Guillain-Barré syndrome in Brazil. PLoS NeglTrop Dis. 2019;13:e0007695. 10.1371/journal.pntd.0007695 PMC6764688 31527907

[11] Yuki N Hartung H-P. Guillain–barré syndrome. N Engl J Med. 2012;366:2294–304. 22694000 10.1056/NEJMra1114525

[12] Paterson RW Brown RL Benjamin L. The emerging spectrum of COVID-19 neurology: clinical, radiological and laboratory findings. Brain. 2020;143:3104–20. 32637987 10.1093/brain/awaa240 PMC7454352

[13] Fokke C van den Berg B Drenthen J. Diagnosis of Guillain-Barre syndrome and validation of Brighton Criteria. Brain. 2013;137:33–43. 24163275 10.1093/brain/awt285

[14] Rajabally YA. Electrophysiological diagnosis of Guillain–Barré syndrome subtype: could a single study suffice?. J Neurol Neurosurg Psychiatry. 2014;86:115–9. 24816419 10.1136/jnnp-2014-307815

[15] Rinaldi S Brennan KM Kalna G. Antibodies to heteromeric glycolipid complexes in Guillain-Barré syndrome. PLoS One. 2013;8:e82337. 24358172 10.1371/journal.pone.0082337 PMC3864991

[16] Klehmet J Märschenz S Ruprecht K. Analysis of anti-ganglioside antibodies by a line immunoassay in patients with chronic-inflammatory demyelinating polyneuropathies (CIDP). Clin Chem Lab Med. 2018;56:919–26. 29329103 10.1515/cclm-2017-0792

[17] Labò N Ohnuki H Tosato G. Vasculopathy and coagulopathy associated with SARS-COV-2 infection. Cells. 2020;9:1583. 32629875 10.3390/cells9071583 PMC7408139

